# Kind Words and Suggestions

**Published:** 1873-06

**Authors:** 


*Kind Words and Suggestions.*

Dr. John H. Wier, of Illinois, writes: "Dr. J. A. Hanks' letter to you was kind, noble and worthy of consideration by every subscriber of the Record, which is a welcome visitor to all who patronize it. And the example of the Doctor should be imitated by all your patrons. I must acknowledge my former negligence. If a little exertion on the part of subscribers was made, a large addition could be made to your list. I here send you a subscriber, and will try and send you more soon."

Dr. J. Knox Hodge, of Arkansas, writes: "Allow us to add that the kind words and suggestions of Dr. J. A. Hanks, of North Carolina, meet our warmest approval. We, for one, are bent on acting on the line indicated in his letter. We trust our fellow-associates will do likewise. We say to our worthy North Carolina brother that the RECORD is *already* 'the best, most useful and the cheapest medical journal ever published in this country.' Let us go to work, with pen and tongue, and make it doubly so."

Dr. G. D. Hodge, of Arkansas, writes: "We will add a hearty amen to the letter of J. A. Hanks, M.D., published in the April number. His suggestion is attended with no difficulty, and as for ourselves, over here in the back-woods, we *will* reach his maximum number of new subscribers, if we have to travel over five counties to obtain them."

The suggestions of our North Carolina friend, Dr. Hanks, are being acted upon, and very soon, we trust, the RECORD will attain the largest circulation of any medical monthly in the country. Dr. Hanks has "fulfilled his promise," and says his "interest in the RECORD has not abated," as he is still working on the same line—of increasing its circulation. If our friends will but emulate his example, our highest expectations will, most fully, be realized. We feel sure there is not one subscriber who may not secure one or two more. We beg them to try.

We have been consulting with a number of medical gentlemen in regard to the best means of promoting medical literature in the South, and of meeting the wants of the profession in the conduct of our journal, and we are gratified to know that thus far our plan and efforts meet the approval of a vast majority of our medical brethren.

In regard to the contemplated enlargement of THE RECORD, there seems to be some difference of opinion, as may be seen by the following response written by one of our associate editors, Dr. R. C. Word, now of Decatur, Georgia:

"We see no necessity for enlarging THE RECORD. If the excellent motto at your masthead—"*Quicquid Prœcipies,*" &c.—is to be observed, then you have space enough. In the main, you have thus far adhered to it, and the profession have been well pleased with the brevity, pith and practical character of your journal.

Southern practitioners—particularly those residing in the country and in the villages remote from our commercial centers—desire, what they have heretofore failed to obtain in the South, a journal practical rather than theoretical, which, while keeping in view a knowledge of diseases as they prevail and are modified by climatic influences in the South, will give, in a *condensed* form, important information obtained from the medical journals of all countries, together with the views of medical men in our own section, presented in such short and practical shape as will keep the reader posted, and enable him promptly to avail himself of all the discoveries and improvements in the profession. The country and village practitioner does not want, and will not read, long, elaborate productions, gotten up to foster the vanity and give notoriety to those who write them. Let those who desire these, seek them in newspapers and quarterlies; but let THE RECORD give us facts, formulæ and brevities culled from the exchanges of the editor, and drawn in pithy original communications from the common-sense practitioners throughout our section. There are thousands of useful and important facts now hidden from the profession which should be made known. There is scarcely a medical man who is not in possession of some valuable fact or discovery developed in his own experience and perhaps known only to himself. It is the duty of all such to report these facts through the journals; and this they should do, whether accustomed to write, or whether able to express themselves in polished language or not. A great and learned philosopher once remarked that he would willingly exchange his knowledge of science and books for the aggregate of all that was known by the unlearned masses of men. We do not go thus far in medicine, yet we doubt not if all the facts known to the comparatively unlearned, yet common-sense practitioners of our land, could be brought to light, that a vast and important accession to medical knowledge would result."

